# Signaling and Adaptation Modulate the Dynamics of the Photosensoric Complex of *Natronomonas pharaonis*

**DOI:** 10.1371/journal.pcbi.1004561

**Published:** 2015-10-23

**Authors:** Philipp S. Orekhov, Daniel Klose, Armen Y. Mulkidjanian, Konstantin V. Shaitan, Martin Engelhard, Johann P. Klare, Heinz-Jürgen Steinhoff

**Affiliations:** 1 Department of Physics, University of Osnabrueck, Osnabrueck, Germany; 2 Department of Biology, Lomonosov Moscow State University, Moscow, Russia; 3 Department of Bioengineering and Bioinformatics and A. N. Belozersky Institute of Physico-Chemical Biology, Lomonosov Moscow State University, Moscow, Russia; 4 Max-Planck-Institute for Molecular Physiology, Dortmund, Germany; Tel Aviv University, ISRAEL

## Abstract

Motile bacteria and archaea respond to chemical and physical stimuli seeking optimal conditions for survival. To this end transmembrane chemo- and photoreceptors organized in large arrays initiate signaling cascades and ultimately regulate the rotation of flagellar motors. To unravel the molecular mechanism of signaling in an archaeal phototaxis complex we performed coarse-grained molecular dynamics simulations of a trimer of receptor/transducer dimers, namely *Np*SRII/*Np*HtrII from *Natronomonas pharaonis*. Signaling is regulated by a reversible methylation mechanism called adaptation, which also influences the level of basal receptor activation. Mimicking two extreme methylation states in our simulations we found conformational changes for the transmembrane region of *Np*SRII/*Np*HtrII which resemble experimentally observed light-induced changes. Further downstream in the cytoplasmic domain of the transducer the signal propagates via distinct changes in the dynamics of HAMP1, HAMP2, the adaptation domain and the binding region for the kinase CheA, where conformational rearrangements were found to be subtle. Overall these observations suggest a signaling mechanism based on dynamic allostery resembling models previously proposed for *E*. *coli* chemoreceptors, indicating similar properties of signal transduction for archaeal photoreceptors and bacterial chemoreceptors.

## Author Summary

Achaea and bacteria can “see” and “sniffle”, they have photo- and chemosensors that measure the environment. On the cell poles, these sensor proteins form large arrays built of several thousands of different receptors. The receptors comprise extracellular or transmembrane sensory domains and elongated homodimeric coiled-coil bundles, which transduce the signals from the membrane across ~20 nm to a conserved cytoplasmic signaling subdomain in an unknown manner. In our study we performed coarse-grained molecular dynamics simulations of the phototactic receptor/transducer complex from *Natronomonas pharaonis*. Comparing fully methylated and demethylated complexes reveals an interconversion between states of different dynamics along the coiled-coil bundle, which might represent the essential characteristics of the signal transfer from the membrane to the binding sites of the downstream kinase CheA.

## Introduction

Phototaxis in archaea is mediated by integral membrane protein complexes consisting of bacteriorhodopsin-like receptors, sensory rhodopsins, and tightly bound transmembrane signal transducers, Htrs. The latter are highly homologous to methyl-accepting chemotaxis proteins (MCPs) in bacteria [1]. Moreover, the archaeal genomes comprise homologs of the principal elements of bacterial two-component systems: the histidine kinase CheA, the response regulators CheY and CheB, and the methyltransferase CheR [2]. This homology suggests that the core properties of signal propagation are conserved and similar in archaeal phototaxis and bacterial chemotaxis [3]. This has been supported by studies of active fusion chimeras of archaeal transducers with bacterial receptors [4,5].

**Fig 1:**
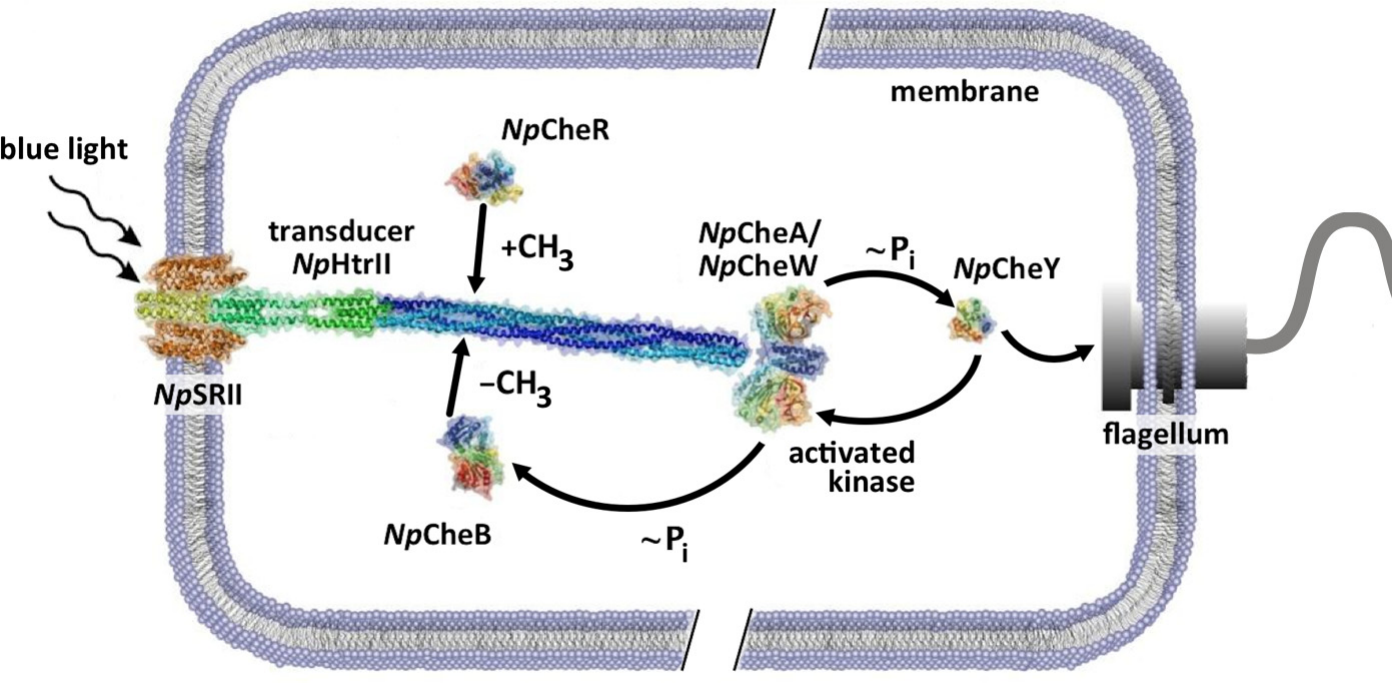
Two component phototaxis system of *N*. *pharaonis*. *Np*SRII/*Np*HtrII dimers are the basic elements of photoreceptor complexes in *N*. *pharaonis*. They consist of two sensory rhodopsins, *Np*SRII, and two transducer proteins, *Np*HtrII, mostly of α-helical secondary structure, with a characteristic domain organization. Light activation of *Np*SRII induces conformational and/or dynamical changes in the transducer which are converted by two HAMP domains and conveyed along the 20 nm long transducer to the tip region, where it activates the homodimeric histidine kinase CheA bound together with the adapter protein CheW. The kinase CheA undergoes auto-phosphorylation and further transfers the phosphate group to the response regulators CheY or CheB. CheY affects the rotational bias of the flagellar motor, while the methylesterase CheB along with the methyltransferase CheR controls the adaptation (feedback) mechanism. The related chemoreceptor and most likely also the photoreceptor dimers further organize into trimers, which, together with CheA and CheW, lead to the formation of large sensor arrays.

The phototaxis system of *Natronomonas pharaonis*, which is composed of sensory rhodopsin II, *Np*SRII, in a 2:2 complex with its cognate transducer, *Np*HtrII, and the Che proteins mentioned above, allows these archaea to avoid harmful blue-green light. It represents one of the best-studied archaeal sensor systems [6] that modulates the cell’s swimming behavior by means of a typical two-component cascade (Fig 1). Light absorption by *Np*SRII leads to activation of the cognate kinase CheA and, ultimately, to alteration of the flagellar rotation mode affecting cell mobility. Light induced conformational changes have been observed for *Np*SRII and *Np*HtrII. Upon photo activation *Np*SRII undergoes an outward motion of helix F [7–10], which induces a clockwise rotation of helix TM2 of *Np*HtrII along with a displacement [8,11,12], presumably similar to the piston-like motion in chemoreceptors [13–15]. These transient changes in the transmembrane part of *Np*HtrII lead to switching between conformational states of the membrane adjacent HAMP domain [16]. At the tip of *Np*HtrII in the vicinity of the CheA interaction site a thermodynamic equilibrium between conformations of different dynamics has been reported [17]. However, the mechanism of signal transduction from the HAMP domains to the tip and the transfer of this signal to the kinase CheA remain to be uncovered.

In bacterial receptors HAMP domains are ubiquitously present separating the transmembrane domain from the cytoplasmic domain. Different models were suggested for HAMP activation and signaling [18,19]. The gearbox model [20] postulates either a relative rotation of helices in the HAMP domain or a combined rotation and tilting motion accounting for signal passage [21]. Alternatively, in the dynamic bundle model [22,23] alteration of HAMP domain dynamics due to a destabilizing effect of the transmembrane region is responsible for signaling. Signal propagation according to the latter model could be related to phase clashes in the heptad repeat pattern of the coiled-coil structure of receptor dimers. A mismatch between characteristic coiled-coil heptad repeats [24] of two domains results in the oppositional dynamic coupling between them: stabilization of the first domain destabilizes the following one downstream [23]. Finally, the possibility of allosteric mechanisms, including signal propagation along coiled coils, driven by dynamics rather than by conformational changes, has been previously discussed [25–27]. Despite the recent progress in the understanding of signaling by HAMP domains, the mechanisms of signal propagation along the cytoplasmic domain as well as kinase activation remain unclear [28]. The former implies changes in the helical packing of the cytoplasmic domain and hereby of its dynamics [29–31]. The latter has been shown to require an organization of the receptors into trimers-of-dimers [32] or even larger assemblies with CheW/CheA [33,34] and might involve local conformational changes in the tip region [35]. Larger arrays of receptors with an extended baseplate of bound CheA and CheW interconnect the trimers and are known to provide signal amplification by means of cooperative activation [34,36,37]. However, the actual mechanism of kinase control by receptor trimers is still unknown, though a recent comparison of receptor/CheA/CheW complexes in different methylation states by cryo-EM tomography indicates that activation of CheA may involve changes in the dynamics of two of the five CheA domains [38].

A reversible methylation/demethylation process of specific Glu/Gln residues located in the cytoplasmic adaptation domain of the receptors [39–41] provides tuning to different levels of input signal intensity at a constantly high level of sensitivity. Two enzymes carry out the modifications, CheR, methylating these sites, and CheB, which catalyzes the competing process of demethylation. The adaptation to the altering signal level is achieved by two mechanisms: activation of CheB by CheA-mediated phosphorylation and opposite propensities for the two modifications in the different methylation states [39]. The major effect of methylation is of electrostatic nature and comprises stabilization/destabilization of the adaptation domain [29]. While particular adaptation systems in other organisms can be more intricate with additional proteins and non-linear effects of intermediate methylation levels on the apparent kinase activity [40,42], it is generally accepted that the two extreme methylation states correspond to different activating states of *E*. *coli* [28] and *B*. *subtilis* [40] chemoreceptors as well as of *H*. *salinarum* phototransducers [41], and consequently to different kinase activity levels, though not necessarily to fully activated or deactivated states.

These parallels between signaling and adaptation processes have been used for investigating different activating states of bacterial chemoreceptors via a change of their methylation level [31,43,44]. The relation between these two processes has also been shown for archaeal photorececeptor/phototransducer complexes [41]. Exploiting the analogy between chemo- and photoreceptors discussed above we have performed coarse-grained molecular dynamic simulations [45,46] to study the effects of methylation/demethylation in trimers of receptor/transducer dimers, *Np*SRII/*Np*HtrII, (referred to as *trimers-of-dimers* further on). In the present study we have modeled differential activating states of trimers-of-dimers as the putatively fully methylated and demethylated states mimicked by charge addition and depletion. The simulations reveal that the removal of negative charges at the putative methylation sites, mimicking methylation [47], causes the adaptation region to adopt a compact and static conformation. The neighboring domains, the kinase-interacting tip and HAMP2, become more dynamic, whereas HAMP1 is again characterized by a static and compact conformation. These alternating dynamics are reversed by demethylation. Our results provide a model for the signaling of archaeal phototactic receptor/transducer complexes at the level of single trimer-of-dimers, that shows striking similarities to those proposed for the *E*. *coli* chemoreceptors [19,23,29–31,48–50]. The results presented here indicate that archaeal phototransducers and bacterial chemoreceptors share a general activation mechanism.

## Results

### Models of the *Np*SRII/*Np*HtrII dimer and the trimer-of-dimers

**Fig 2:**
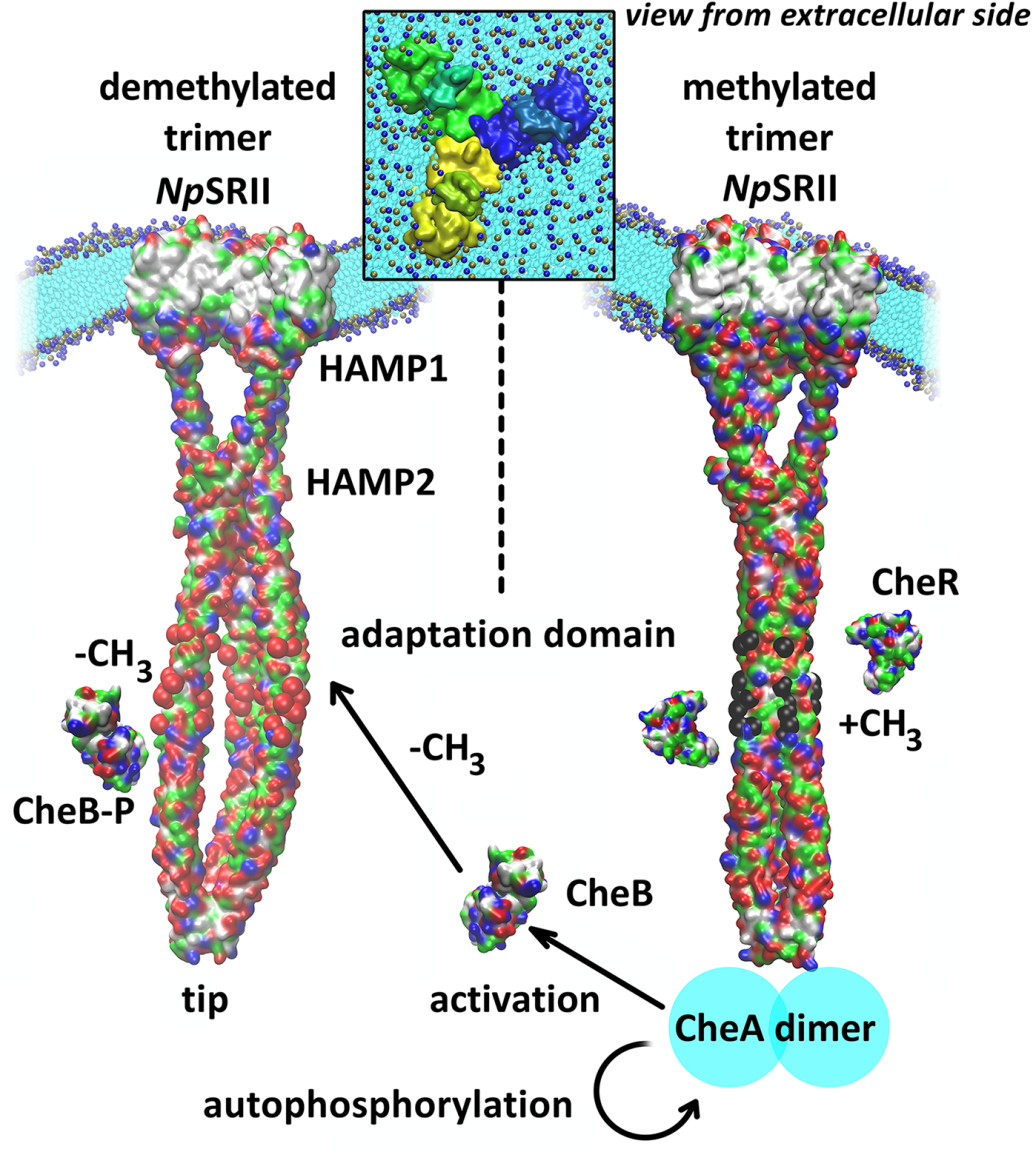
Conformations of the trimeric photoreceptor-transducer complexes. Cartoon with the resulting structures of the demethylated (left) and the methylated (right) trimer systems combined in the bent membrane with a schematic representation of the adaptation process. Methylation sites are shown as red and black spheres in the demethylated and methylated state, respectively.

To reveal structural and dynamical effects of methylation/demethylation of the *Np*SRII/*Np*HtrII complex we have built a coarse-grained model of trimer-of-dimers based on a pre-equilibrated all-atom model of the 2:2 *Np*SRII/*Np*HtrII complex. The latter has been assembled by combining the crystal structure of the transmembrane part of *Np*SRII/*Np*HtrII with structures based on homology modeling as described in the *Methods* section (see S1 Fig). The model dimer embedded in a model *E*. *coli* lipid membrane was equilibrated for in total 500 ns of all-atom MD until the root mean square deviation (RMSD) of the whole structure became stable. The equilibration led to two changes in the dimer structure (see S1 Fig). First, the inter-HAMP region rapidly formed an asymmetric coiled-coil with one helix shifted with respect to the other by approx. 1.4 Å (see S2 Fig). This shift caused a tilt of the whole dimer and corresponds to the minimum of the free energy for the isolated inter-HAMP region [51]. Second, both sensory rhodopsins underwent a motion in the transmembrane region resembling the U-V shape transition observed in recent X-ray structures [12]. This rearrangement preserves most contacts between *Np*SRII and *Np*HtrII and could provide a route for receptor cross-talk in the dense membrane lattice which might be physiologically relevant. Subsequently, we have constructed a model of the trimer-of-dimers as documented in detail in *Methods*. Briefly, using an appropriate snapshot from the dimer equilibration, we assembled a trimer-of-dimers based on the X-ray structure of the bacteriorhodopsin trimer (pdb code 2NTU [52]). Due to the large system size of the complex of ~323,000 atoms, the all-atom model was subsequently converted into a coarse grain (CG) representation [45] and embedded into a CG-POPC bilayer (S1 Fig). The solvated CG model of the methylated trimer-of-dimers was equilibrated for 2 μs with constraints on the inter-dimer interface in the highly conserved tip region (positions 340–380) known from both X-ray crystallography (pdb code 1QU7) [53] and NMR studies [54]. In a subsequent 6 μs equilibration step without any constraints the inter-dimer interface contacts remained stable. The equilibration simulation was followed by repeated production simulations of 2 μs each for the methylated and the demethylated systems (the latter had been first equilibrated starting from the final structure of the unconstrained equilibration simulation of the methylated system until convergence of the measured observables was achieved in 2 μs, see Methods). During the equilibration the *Np*SRII/*Np*HtrII trimer induced a pronounced membrane curvature, which we quantified for both the methylated and the demethylated systems along two perpendicular directions within the membrane plane. As shown in Fig 2, the membrane was highly and equally bent with average radii of curvature of 99.4±0.2 Å and 99.3±0.2 Å for the methylated and the demethylated systems, respectively. As indicated by the small standard deviations, the curvature radii in both systems do not change remarkably throughout the trajectories (S3 Fig).

### Conformational rearrangements upon adaptation

**Fig 3:**
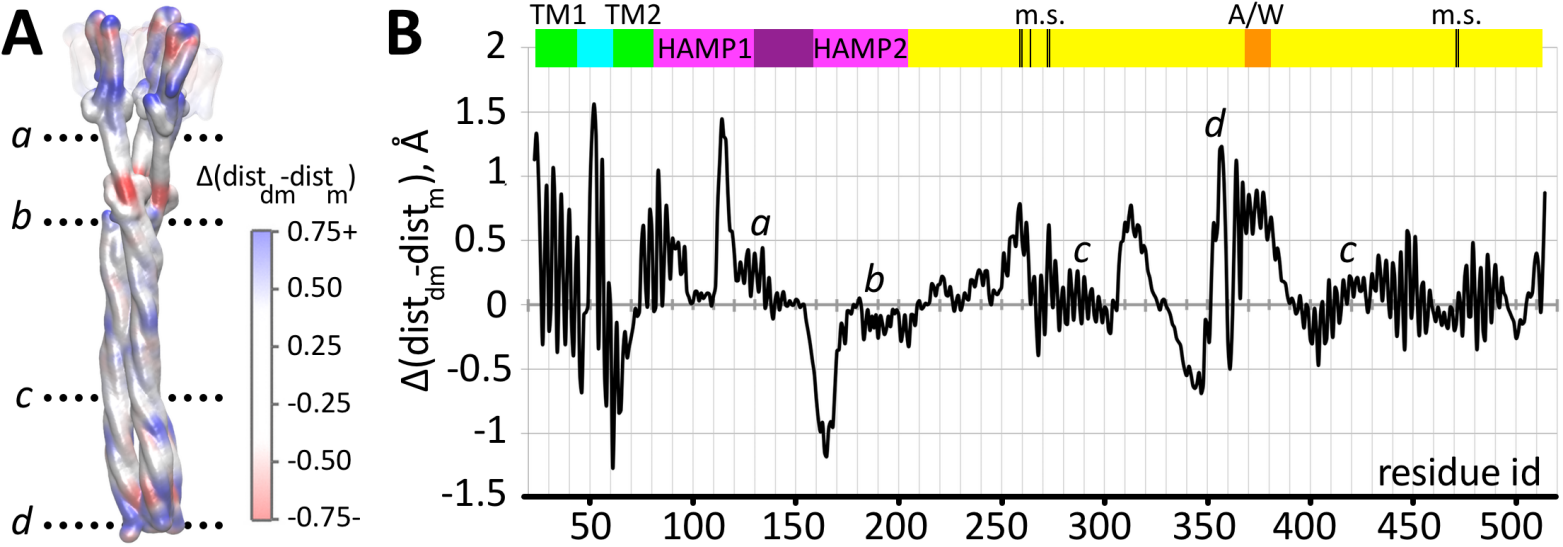
Intra-dimer distance changes between related residues of the transducer upon demethylation A: Structure of the *Np*SRII/*Np*HtrII trimeric complex with distance changes color coded (calculated as an average over the three dimers). Positive values of the distance difference (blue) indicate a looser packing of the corresponding residues in the demethylated system, negative values (red) indicate a more compact packing. B: The intra-dimer distance difference as function of residue number shows distinct changes in the transmembrane region of the complex, an inversion of the packing densities for the two HAMP domains, minor changes at the methylation sites (m.s.), and a decrease of the packing density in the CheA/CheW binding site region labeled A/W.

**Fig 4:**
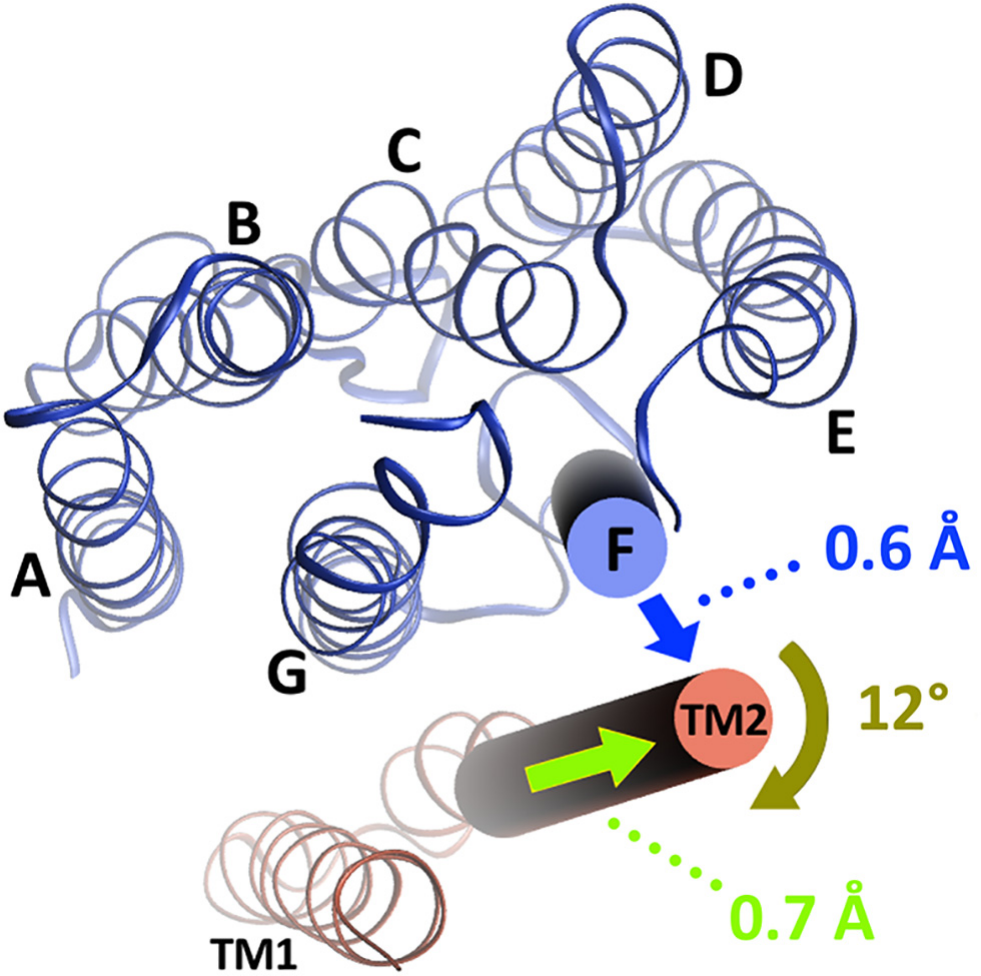
Comparison of the conformational changes of the transmembrane region upon demethylation and illumination. Cytoplasmic view of a monomer of the *Np*SRII/*Np*HtrII complex with experimentally observed conformational changes upon illumination shown as arrows and corresponding numerical values from the CG MD simulations. An outward tilt of helix F at the cytoplasmic side of the membrane embedded part of the transducer by 0.6±0.3 Å (blue arrow) is accompanied by a rotation of helix TM2 of 12±8° (olive green arrow) with respect to the equilibrated methylated structure. In addition TM2 shifts with respect to the helix TM1 of the transducer by 0.7±0.5 Å (light green arrow).

Possible structural rearrangements within the *Np*HtrII dimers upon demethylation/methylation mimicked via charge reshaping in the adaptation region are analyzed in terms of the inter-backbone distances (Fig 3). Distance changes are most prominent in the membrane embedded part, in the two HAMP domains and in the CheA/CheW interaction region. We also compare the effects of methylation and demethylation with those induced by light activation. Fig 4 summarizes this comparison for the membrane embedded part of the phototaxis receptor/transducer complex demonstrating that methylation causes similar effects as observed experimentally upon illumination: The outward motion of helix F of *Np*SRII observed in the simulation is in agreement with the experimentally observed tilt triggered by the photoinduced *cis-trans* isomerization of the *Np*SRII retinal chromophore [7,8,10] (for details of the trajectories see S4 Fig). The coupled transducer helix TM2 rotates by approx. 10–15° as shown experimentally by EPR spectroscopy [8] and by X-ray crystallography [11]. This rotation is accompanied by a piston-like motion of the TM2 helix by approx. 0.5–1 Å as also seen in the crystal structure [11]. Most intriguing, a recent study revealed an influence of the *Np*HtrII signaling domain on the *Np*SRII photocycle kinetics [17], providing additional experimental evidence for the conformational coupling of receptor and transducer in these complexes. The agreement between light induced conformational changes for the transmembrane domain observed in the experiments and our simulations corroborate the present approach and calculations and gives confidence to the results obtained for alterations in the cytoplasmic domain.

The opposite differences in the intra-dimer helix distances obvious for the two HAMP domains suggest that their packing is coupled in structural opposition (Fig 3). In the methylated state packing of HAMP1 is tighter whereas for HAMP2 it is significantly looser. This is in agreement with the dynamic-bundle model [18,23] according to which a phase stutter connection between the HAMP and the downstream bundles couples their packing stabilities oppositely in structurally adjacent segments. These phase stutters coincide with discontinuities in the coiled-coil heptad repeats [23] between HAMP1 and HAMP2 and between HAMP2 and the methylation sites (see S5 Fig). Tight packing of the HAMP1 helices is thus coupled to a loose packing of HAMP2 and to a tight packing of the downstream bundle helices, and *vice versa*. Strikingly, the transition between methylation and demethylation does not lead to gross changes of the backbone packing density in the adaptation region (labeled m.s. in Fig 3). In contrast, larger differences in the intra-dimer backbone distances are again revealed in the regions which include the sites responsible for the interaction with the kinase CheA [54]. Here most prominent inter-backbone distance changes span more than 40 residues along the N-terminal (positions 310–350) and nearly 20 residues along the C-terminal part of the tip (positions 367–390). This conformational rearrangement must result in a reorganization of the respective side chains which is beyond the resolution of the present method but may be the signal propagated to the bound kinase CheA. The inter-backbone distance change in the very tip region of the transducer including the helix turn (positions 352–365) oscillates with negative as well as positive values which is evidence that the tip structure as a whole remains intact. A calculation of the relative inter-helical shifts in the tip region did not show any sliding upon demethylation; neither between the helices within one monomer nor between the two monomers in a dimer (see S6 Fig), in accordance with a previous experimental study [55]. This is strong evidence that the tip presents stable interaction sites for association with the kinase proteins as previously suggested [56].

**Fig 5:**
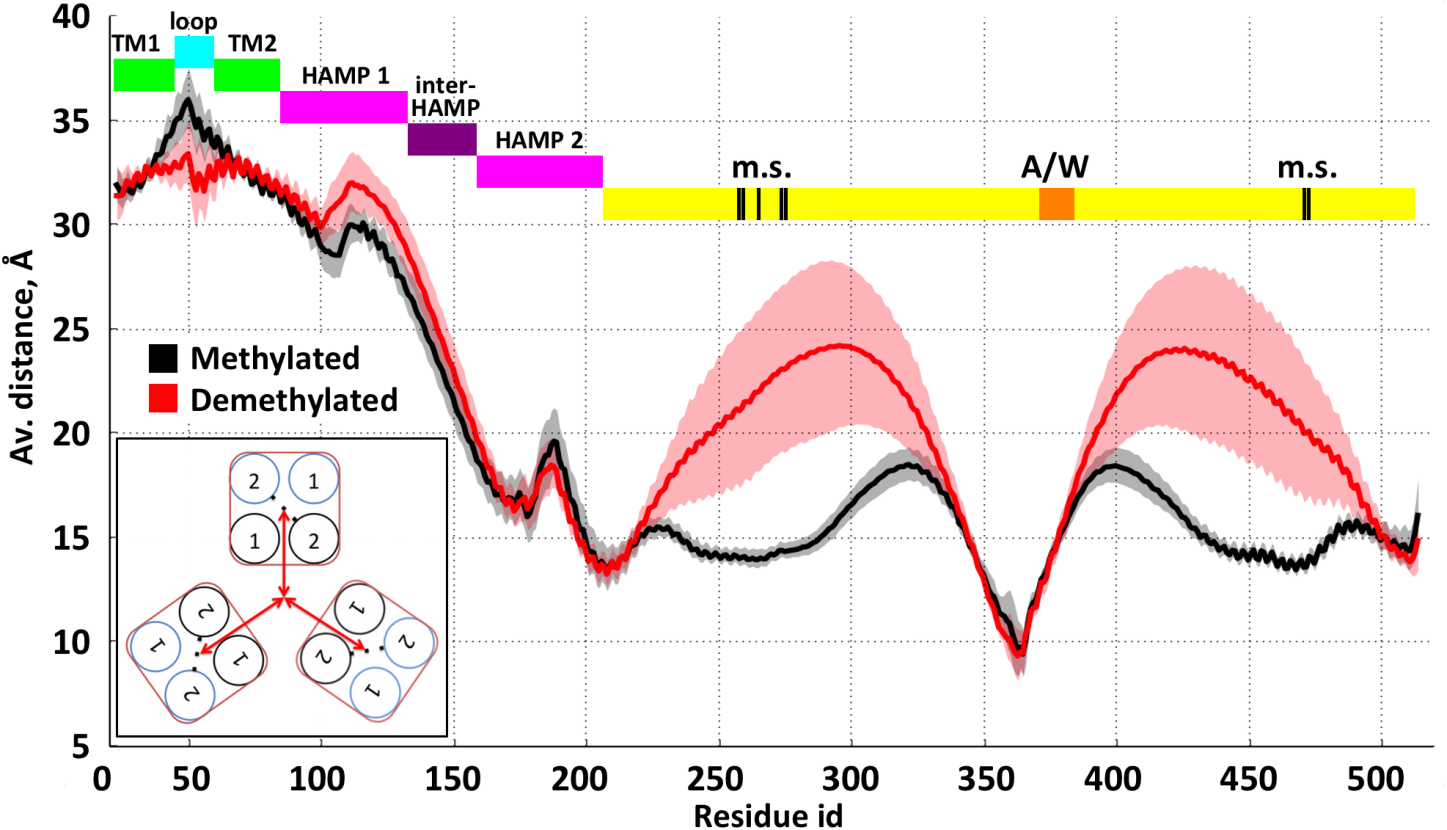
Inter-dimeric distances for related residues of the transducer. Distances were calculated as an average over the three dimers for the methylated (black) and demethylated (red) states, shaded areas representing the standard deviation. The distance is measured between the center of mass (COM) of two related residues in one dimer and the COM of the six respective residues in the trimer-of-dimers (see inset on the lower left). The domains of the complex are depicted in colored bars; m.s and A/W indicate methylation sites and binding sites for CheA/CheW, respectively. Representative distance trajectories are depicted in S7 Fig.

Methylated and demethylated trimer structures differ substantially in their conformations (Fig 5). The average distances between each dimer and the trimer central axis are primarily affected in the adaptation region as well as in both HAMP domains. In the adaptation region the additional charges generated by demethylation lead to strong electrostatic repulsion between the dimers causing deviations in the inter-dimer distances of up to 10 Å (Fig 5, S7 Fig). The first and second HAMP domains show an inverse response in their inter-dimer distances (Fig 5, S7 Fig). In contrast, inter-dimeric distances in the tip region do not significantly change (S7 Fig). In spite of the observed detachment of the dimers in the adaptation regions, the total length of the complex did not change upon demethylation (S8 Fig). Thus, the conformational rearrangements of the trimer observed upon methylation do not appear to cause a major change of the trimer conformation at the tip, which again corroborates the notion that the tip is a stable structural unit for interaction with the CheA/CheW kinase platform.

In conclusion, intra- and inter-dimer conformational rearrangements are obvious upon demethylation. These conformational rearrangements are found to be in agreement with experimental data characterizing conformational changes of the transmembrane domains upon light activation [8,11,57]. The results are further in line with the prediction of the dynamic-bundle model and reveal conformational changes in the region of the CheA binding sites. The propagation of the signal along the cytoplasmic bundle to the tip, which is not obvious from the structural characterization, will be uncovered in the next section.

### Signal transduction between the transmembrane domain of the transducer and the cytoplasmic domain

The picture of the structural rearrangements in the *Np*HtrII/*Np*SRII trimer-of-dimers is incomplete without the description of the structural changes conveying the signal between the adaptation region and the transmembrane domain of the complex, where the conformational changes were found to resemble the light induced changes. The analysis of the CG MD trajectories points to an opposite relative longitudinal shift of the two helices in the inter-HAMP region as a putative structural factor of the observed dynamics conversion between the two HAMP domains (S9 Fig and S10 Fig). The plausible role in signaling of this inter-HAMP region was previously highlighted by Gushchin and coworkers [51]. On the other hand, the large difference in the inter-dimer helix distances within the region connecting the transmembrane domain of the transducer and the HAMP1 (Fig 3) implies the presence of decisive structural alterations in this region between the methylated and the demethylated systems. This may have parallels with the proposed change of the control cable helicity in chemoreceptors upon the kinase-off to kinase-on transition [58].

### The adaptation process alters the dynamics of the complex

**Fig 6:**
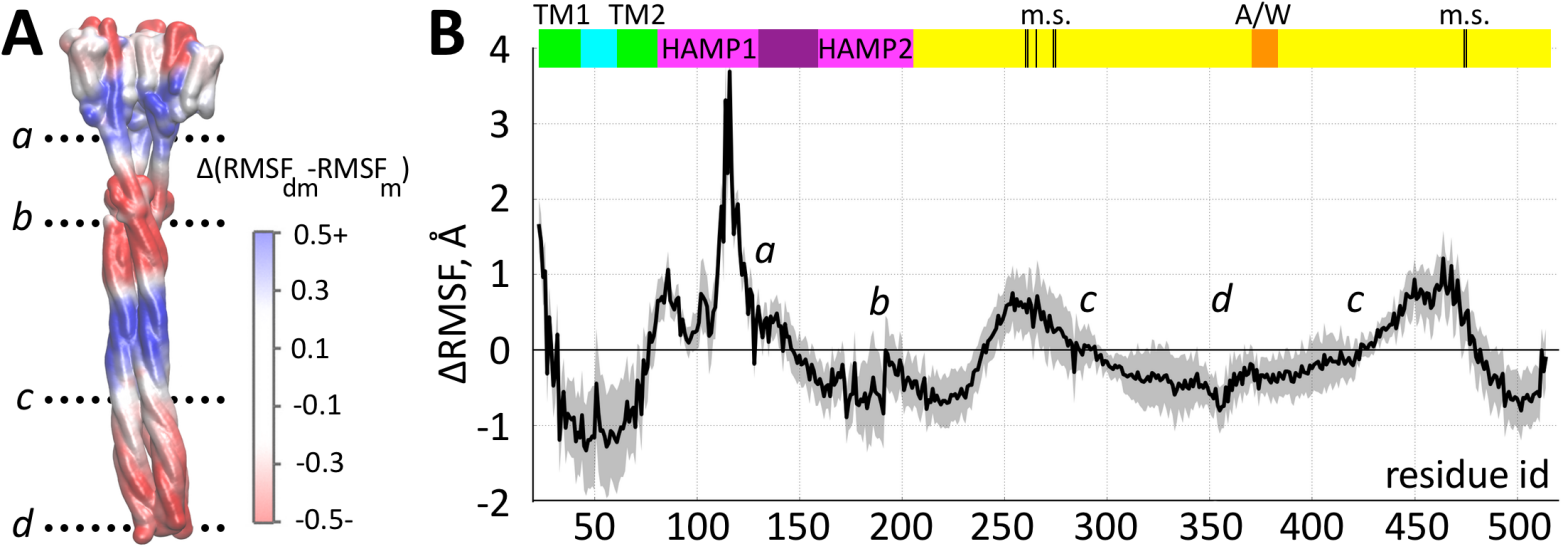
Dynamics of the methylated and the demethylated systems. A: Structure of the *Np*SRII/*Np*HtrII trimeric complex with colors that code for the difference between the RMSF value per residue of the demethylated and the methylated transducer. Positive values (in Å) correspond to a higher fluctuation and therefore higher mobility of the corresponding residues in the demethylated system, negative values indicate a lower mobility. B: The differences in mobility as function of residue number show distinct changes in the transmembrane region of the complex, an inversion between the two HAMP domains and in the adaptation and close to the glycine rich (293, 296) regions. This change in dynamics upon adaption includes the tip region and the binding sites for CheA (A/W). Colored bars have the same meaning as in Fig 3.

Adaptation clearly affects the dynamical characteristics of the complex. Analyses of the difference in the root mean square fluctuations (RMSF) per residue between the demethylated and the methylated complexes reveals regions with an alternating sign of ΔRMSF (Fig 6). In the demethylated state zones corresponding to the first HAMP domain and the adaptation region show higher mobility than in the methylated system, while zones of the transmembrane region of the complex, the second HAMP domain and the tip region exhibit lower mobility.

Earlier experiments have shown that the first HAMP domain of *Np*HtrII is engaged in a thermodynamic equilibrium of two conformations, a dynamic and a more compact state [59]. Light activation of *Np*SRII and the propagation of the corresponding signal *via* rotation of helix TM2 shift this conformational equilibrium towards a more compact conformation [57]. This shift was found to be of opposite sign in HAMP2 [60] (L. Li and M. Engelhard; C. Rickert et al.; unpublished), making this domain more dynamic upon light activation. These observations are consistent with the sign inversion of the fluctuation differences observed here for the HAMP1 and HAMP2 domains (Fig 6).

Again, the boundaries between two zones with different ΔRMSF coincide with discontinuities in the coiled-coil heptad repeats, termed phase stutters [23], between HAMP1 and HAMP2 and between HAMP2 and the methylation sites (Fig 6 and S5 Fig). The change of the dynamic pattern observed between the methylation sites and the tip is located close to the glycine hinge region (positions 293, 296) previously recognized as a structural element important for signal propagation [61]. This dynamics change is correlated to changes in the geometry of helix interaction: In the methylated state the helix conformation switches from a knobs-into-holes (“da”) residue packing to a complementary “x-da” packing close to position 240, where ΔRMSF changes from negative to positive values, and back to “da” packing in the glycine hinge region (293, 296), where again ΔRMSF changes sign (see also S11 Fig). These two packing states, related by axial rotation of the helices, were proposed to be associated with different signaling states of HAMP [20] and adaptation domains [56] in chemoreceptors.

This observed pattern of dynamics is distinctly different from a globally altered flexibility, which would merely lead to a different extent of Brownian motion around the membrane anchor of the trimer. The observed domain-specific altered flexibilities reveal a tight control of the local dynamics in the coiled-coil transducer structure. In between the second HAMP domain and the CheA interaction sites close to the tip region, the small inter-helical distance changes (Fig 3) indicate close structural similarities on the backbone level in the two states, while the dynamics (Fig 6) clearly depend on the methylation state. Therefore, signal propagation via different dynamical states seems to provide the link between the CheA-activating region and the membrane-proximal HAMP domains.

## Discussion

To investigate signal transduction in archaeal phototaxis complexes we have studied a trimer-of-dimers model of the *Np*HtrII/*Np*SRII photosensoric complex exploiting the analogy (see Introduction) between bacterial chemoreceptors and archaeal phototaxis signal transducers as well as the relation between activation by native stimuli and adaptation established experimentally for chemoreceptors [3–5,31,41,43,44,47,62].

As it is the charge of the amino acid side chains undergoing methylation, rather than their size or shape, that modulates kinase activity [29] [30], the effect of methylation and demethylation was mimicked by varying the charges of these amino acid positions. In the transmembrane region activation by light is known to result in a characteristic tilt of helix F of *Np*SRII [7,9] and a rotation of helix TM2 of the transducer *Np*HtrII [8,11] possibly accompanied by a piston-like motion [11,60]. Intriguingly, in our simulations the structural differences observed in the transmembrane part for the methylated and demethylated trimer resemble the experimentally determined behavior in response to light activation: methylation rotates TM2 by 10–15° and shifts it by 0.5–1 Å (Fig 4). In addition an outward movement of helix F of the *Np*SRII is revealed (Fig 4). In the HAMP domain region, activation by light leads to a more compact conformation of HAMP1 and equivocally to a more dynamic HAMP2 domain [57,59] similar to the changes observed in the present simulations (Fig 4). These observations support the conclusion that, at least for the transmembrane part of the *Np*SRII/*Np*HtrII complex and its HAMP domain regions, the mechanism for the signal propagation upon light activation strongly parallels the simulated changes by adaptation exerted through the methylation/demethylation of the transducer.

Our results indicate that the cytoplasmic tip of the trimer does not undergo a gross structural rearrangement when methylation or demethylation occurs, in spite of the observed large scale opening of the adaptation domains of the dimers caused by electrostatic repulsion. Interestingly, the total length of the complex does not change significantly. Thus, the interface between the tip and the baseplate of CheA/CheW proteins remains largely intact, which might be an important factor for the integrity of signaling arrays. However we are aware of the fundamental limitation of the present model of the trimer-of-dimers, i.e. the omission of the baseplate proteins. Further computational and experimental studies are required to elucidate the role of the receptor-baseplate contacts and their dynamics in signaling.

**Fig 7:**
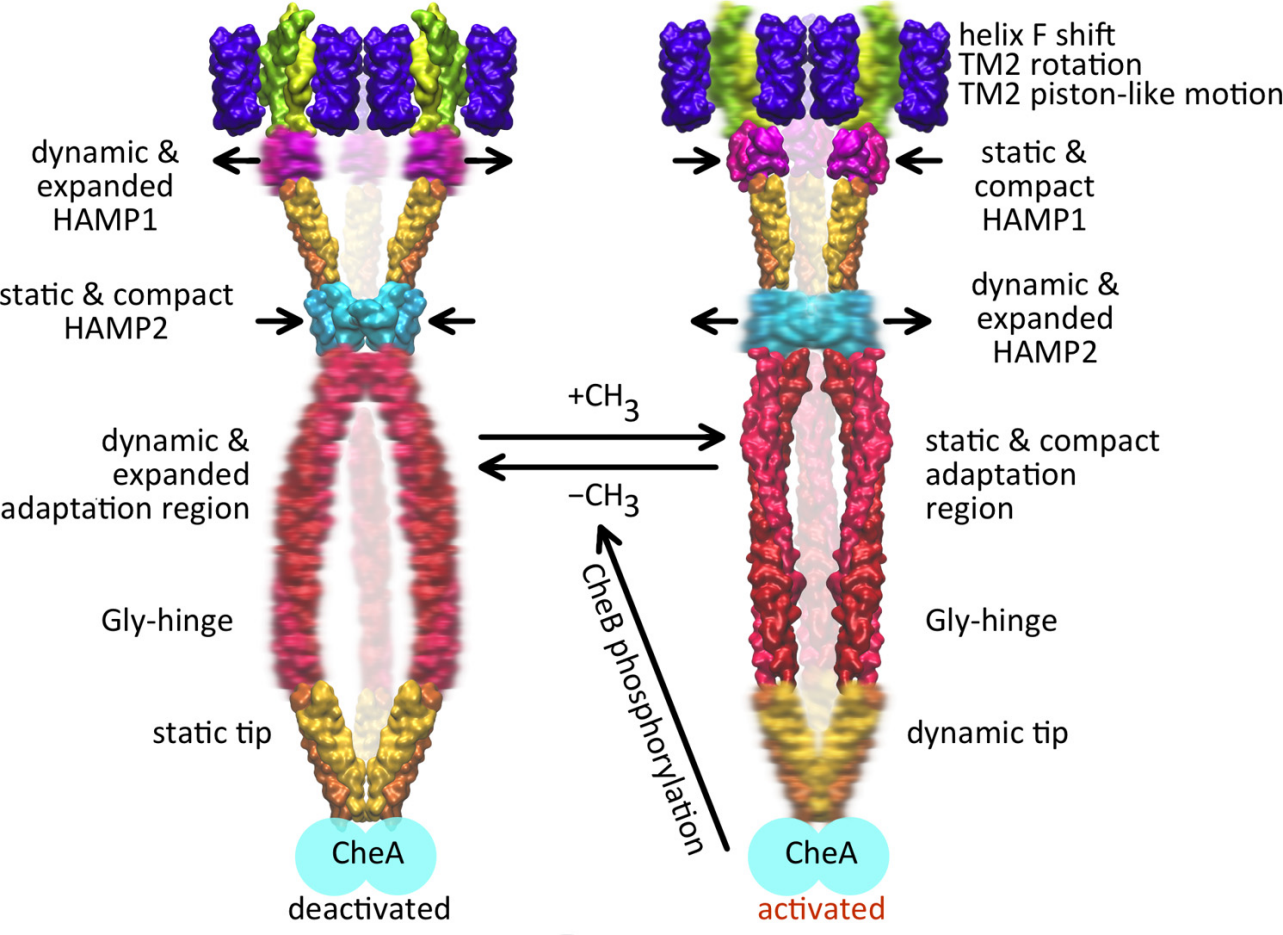
Model for the *Np*SRII/*Np*HtrII complex activation. The regions with higher mobility are shown in diffuse representation; the arrows correspond to the domain motions (compacting/expanding within the trimer).

In contrast to the lack of conformational rearrangements in the tip discussed above, the dynamics show distinct differences between the methylated and demethylated states all over the transducer trimer. Demethylation induces a more dynamic behavior of the adaptation domain but leads to less dynamics of the tip (Fig 6). The two HAMP domains also respond in opposite ways, with HAMP1 adapting a looser dynamic state while HAMP2 adapts a more compact and static conformation. In the methylated trimer this pattern is inversed: static HAMP1—dynamic HAMP2—static adaptation domain—and dynamic tip. Notably, the glycine-rich hinge region seems to constitute the interface between methylation sites and tip and separates the two zones with different mobility (Figs 6 and 7). This dynamic pattern is coupled to structural rearrangements. Generally, the intra-dimeric distances (Fig 3) increase in those regions of the trimer which experience a more dynamic behavior (Fig 6), and the changes in packing density are accompanied by changes in the axial rotation states (S11 Fig) as observed for different crystal structures of methyl-accepting domains [56].

A relation between signaling or adaptation and receptor dynamics as observed here for the phototaxis receptor complex has already been reported for chemoreceptors. B-factor analysis of X-ray structures of *E*. *coli* Tsr shows that methylation exerts a stabilizing structural influence by driving the HAMP domain towards a more compact and less dynamic state [43]. Also mobility changes of residues were observed upon signaling in chemoreceptors [43,63,64]. In particular, a yin-yang model has been proposed as a conceptual basis for signal transduction in chemoreceptors [30]. In this model a coupling region transmits each change in helix packing from the adaptation region to the tip, where it triggers a change in helix packing that is concerted but opposite in sign. Based on experiments [30] local transitions between a tightly packed, less mobile, “frozen” conformation and a more loosely packed, dynamic conformation of the adaptation domain were found rather than a global dynamical transition for the whole receptor as was suggested in [50]. Our findings are in agreement with and provide further evidence for this yin-yang model and show that the tip region undergoes a dynamic–“frozen” transition rather than a local conformational change between two relatively stable states. This model does not exclude the important role of the previously found conformational switches in the tip region [35] and of the HAMP1 domain [65] in the modulation of such dynamical behavior.

In conclusion, the results from our CG calculations demonstrate that the dynamics of the archaeal transducer *Np*HtrII are modulated by methylation/demethylation and, likely, photostimulation in a cassette-like manner. The alternating differences in dynamics, which are characteristically coupled to structural rearrangements, are propagated to the kinase interactions sites. Thus, the coin of signal transmission along the rod-shaped cytoplasmic domain is represented by consecutive subdomains finely tuned by a cascade of alternating dynamics.

The question remains how the signal is transferred from the transducer trimer to the kinase CheA, as we omitted the baseplate proteins in the current study. However, we can speculate here that a gross alteration of the interaction between the transducer tip and the CheA/CheW baseplate seems to be unlikely [56]. The observed changes of the local coiled-coil backbone packing of the transducer in the CheA-interaction region may alter the transducer surface epitope, which could propagate a local conformational change via the transducer—CheA interface and thereby modulate kinase activity. However, complete dissociation of the high-affinity binding interface has been excluded [66]. The other possible scenario might comprise a change in CheA dynamics that can be propagated within the five domains and consequently affect the internal dynamics of CheA. Evidence for this hypothesis stems from the importance of the hairpin residue flexibility situated on the tip of Tsr receptors [67]. A signaling mechanism based on an altered dynamics may also explain recent findings from cryo EM and proteolysis susceptibility experiments [38] which show that activation of CheA by Tsr leads to higher mobility of P1 and P2 domains of CheA. P1 and P2 carry the phosphorylation site and are responsible for CheY and CheB binding, respectively. Such scenario of a dynamical modulation of the CheA activity may also account for the signal spread across neighboring core complexes [28]. Similar models of dynamical allostery have been reported before for different systems [27,68].

CG molecular dynamics were essential to build and equilibrate this large system as well as to study the general mechanism of methylation and signal propagation. For a more detailed view of changes in side chain interactions within and between the involved proteins that give rise to the dynamical signaling, a more detailed investigation by all-atom molecular dynamics is required. Additionally, dissecting the role of dynamical and conformational changes of the transducer and CheA in a methylation state-dependent fashion will allow to distinguish different mechanisms of CheA activation. To this end, EPR spectroscopy on spin labels engineered into the transducer tip or the linkers between the five CheA domains could probe for effects of signal propagation. Alternatively, aiming for a fully detailed view, NMR spectroscopy combined with relaxation dispersion experiments can provide structural and dynamical information on the micro- to millisecond timescale, and show how the linker of the isolated CheA-P4 domain influences the phosphorylation activity of CheA [69].

The results reported here for the two extreme methylation states of the trimer-of-dimers suggest a mechanism for signal propagation in archaeal photoreceptor-transducer complexes similar to that in bacterial chemoreceptors. Accordingly, upon light activation of *Np*SRII the signal is transferred to *Np*HtrII via a movement of helix F and a concomitant screw-like motion of TM2. The latter conformational change leads to alternating dynamics of HAMP1, HAMP2, the adaptation domain, and the CheA binding sites of the transducer. This mechanism is substantiated by experimental evidence such as rotary movement of TM2 [8] and observations concerning the dynamic pattern of the cytoplasmic domain [16,17]. The proposed mechanism provides an explanation how the seemingly subtle input stimulus actuated in the transmembrane part of the receptor can pass over its whole length and eventually affect the activity of the kinase bound on the opposite extremity of the receptor complex, which is 260 Å apart. The observation of subdomains along coiled-coil structural elements which can alter their structural and dynamical properties might be the basis for a universal mechanism in these structural elements not only found in chemo- and phototaxis.

## Methods

### Preparation of the models of the dimer and the trimer-of-dimers

An all-atom (AA) model of the *Np*sRII/*Np*HtrII dimer was prepared based on the available structures of different domains of the complex. The X-ray structure from 1H2S was used as a starting point for the transmembrane region of the complex (consisting of the *Np*sRII dimer and the part of transducer from Gly 23 to Leu 82), while models generated by homology (the modeling scores are provided in S1 Table) with the available structures were utilized to build the cytoplasmic region consisting of the two HAMP domains (template: NMR structure 2ASX from *Archeoglobus fulgidus*) and the cytoplasmic domain (template: X-ray structure 2CH7 from *Thermotoga maritima*) using Modeller [70]. The connecting region between the first and the second HAMP domains was predicted as a coiled-coil α-helix, and it was modeled as an ideal α-helix [51,71]. All the structures were aligned using the helical overlap between the adjacent domains.

The model was embedded into a lipid bilayer containing 75% POPC and 25% POPG, which resembles a typical prokaryotic lipid composition using the CHARMM-GUI service [72]. After solvation and addition of Na^+^ and Cl^-^ (to neutralize the system at a salt concentration of 0.15 M) the system contained 323,096 atoms in total.

This all-atom model of the dimer in a prokaryotic model lipid membrane was subjected to an extensive equilibration simulation (See main text and S12 Fig for RMSD plots).

To construct a model of the trimer-of-dimers we took a snapshot from the dimer equilibration trajectory with the shift of the cytoplasmic domain securing an adequate mutual orientation (i.e., no overlaps) of the dimers within the trimer. The model of the trimer itself was assembled using the X-ray structure of the bacteriorhodopsin trimer (PDB code 2NTU) [52] as a template for the structural alignment. We have chosen this relative orientation of the transmembrane domains of the dimers within the trimer-of-dimers based on the oligomer conformations predicted with the help of the protein-protein docking software M-ZDOCK [73,74]. Two oligomer conformations for the transmembrane part of the trimer-of-dimers were obtained (S13 Fig), with the top-ranked one (“triangle”-like) featuring the inter-rhodopsin contacts similar to those in the bacteriorhodopsin trimer. The second ranked alternative conformation (“ring”-like) (S13 Fig) significantly differs within the transmembrane region of the complex but not in the cytoplasmic domain. According to these results we preferred to study the “triangle”-like conformation because its conformation corresponds to established inter-protein contacts. One *Np*SRII monomer of each *Np*SRII:*Np*HtrII dimer was aligned with one of the three monomers within the bacteriorhodopsin trimer using the Chimera [75] Match & Align tool (combining sequence and 3D structures alignment).

The built all-atom model was embedded into a lipid bilayer containing POPC using the VMD [76] Membrane plugin and subsequently converted into the Martini coarse grain (CG) representation [45]. This CG model of the trimer-of-dimers was placed into a simulation box filled with CG water particles and ions (each resembling 4 water molecules, the system was neutralized at a salt concentration of 0.15 M, the total number of particles equaled 155,747) and equilibrated for 2 μs with the tip region first steered toward the known X-ray interface (PDB code 1QU7 from *Escherichia coli*) [53] and then constrained with the interface contacts preserved. This constrained MD simulation was followed by a further unconstrained equilibration of 6 μs. A number of production simulations were performed as listed in Table 1 after the equilibration simulations. The equilibration simulation of the demethylated system was started from the resulting structure of the unconstrained equilibration run of the methylated system.

**Table 1:** List of simulations performed.

System	Model	Duration, repetitions
Equilibration of the dimer	AA	0.5 μs
Equilibration of the methylated trimer (constrained)	CG	2 μs
Equilibration of the methylated trimer (unconstrained)	CG	6 μs
Production run of the methylated trimer	CG	2 μs x 2
Equilibration of the demethylated trimer	CG	2 μs
Production run of the demethylated trimer	CG	2 μs x 2
Demethylated-to-methylated transition run	CG	1 μs

We performed an additional simulation starting from the equilibrated demethylated system, in which the methylation state was swapped to the fully methylated one. Over the course of this simulation we observed structural changes similar to the previously found differences between the methylated and the demethylated systems. In S14 Fig the time evolution of these changes during the demethylated-to-methylated transition is shown. These additional results proof statistical significance of our observations and indicate that the simulated systems are not trapped in local potential minima but explore the available phase space.

Trajectories were produced with a total of 19 μs of CG time, in which sampling is 3–6 times faster compared to all-atom simulations due to the relative smoothening of interactions [77].

### Molecular simulations

Classical molecular dynamics simulations were performed in the Gromacs 4.5.3 software package using the CHARMM36 forcefield [78,79] with CMAP corrections and the water model TIP3P [80] at ~150 mM NaCl ions to neutralize the system. Control simulations with 3 M NaCl resulted in an equal *Np*HtrII conformation in terms of its RMSD and RMSF values. For the retinal chromophore the set of parameters from [81] was used. The NPT ensemble was maintained with a Parrinello-Rahman barostat (semi-isotropically with compressibility equals 4.5∙10^−5^ bar^-1^) and Nose-Hoover thermostat (323 K, τ_t_ = 2 ps). The cut-off for electrostatic interactions was set to 1.2 nm, while the long-range electrostatics was treated with PME. The time step of 2 fs was used for the all-atom simulations.

Since large secondary structure alterations are expected not to occur during the signal propagation through *Np*HtrII [16,17,30,57], we employed the MARTINI CG model, which is adjusted for a description of protein-protein interactions rather than for secondary structure formation or changes [82]. The standard MARTINI protocol was used for CG simulations in Gromacs as introduced in [45,83,84]. The retinal in *Np*SRII has been omitted without loss in stability of the receptor (see S15 Fig for RMSD plot), and the common MARTINI approach for atoms-to-particles mapping was used with an average ratio of 4:1. Inter-particle Lennard-Jones interactions are described in MARTINI in a form of four basic types of interacting particles (polar (P), charged (Q), mixed polar/apolar (N) and hydrophobic apolar (C), depending on their polarity or capability for H-bond formation) subdivided further into 18 additional subtypes all interacting at 10 different levels (from supra attractive 0 –through intermediate IV–to super repulsive IX). Electrostatics is treated in MARTINI according to Coulomb’s law based on the partial charges assigned in the force field. Protein secondary structure is preserved with constraints imposed to the regions with α-helical secondary structure, while coil regions are treated unconstrained allowing flexible regions of the complex to adjust their tertiary conformation. Though constraining of the structure by means of elastic network restraints could be used, we did not apply any additional restraints in the production simulations.

In the course of the trimer-of-dimers assembling additional constraints were imposed to steer the tip regions of the three dimers towards the contacts established experimentally [53]. As a target for the steered and constrained MD simulations a homology model of the highly conserved tip region of the trimer-of-dimers was derived from the X-ray structure of the trimer-of-dimers (PDB code 1QU7 [53]). Steered MD module of the PLUMED 1.3 plugin [85] was used to carry out these steered simulations using a harmonic potential with a force constant of 1000 kJ/(mol nm^2^).

To identify possible methylation sites we built a sequence alignment (S2 Table) for *Np*HtrII and two chemoreceptors for which the methylation sites were found experimentally, the Tsr receptor of *E*. *coli* [53] and TM1143 from *Thermotoga maritima* [86]. According to their homology or location with respect to these sites seven amino acid positions were admitted as putative methylation sites, namely Q259, Q260, E264, E273, E274, E469 and Q470. In addition, the sequences containing the residue pairs E273, E274 and E469, Q470 are identical or similar to the transferase recognition consensus sequence, respectively (see S2 Table). Q259, Q260 correspond to Tsr positions E274, E275. E264 is located close to identified positions E281(Tsr) or Q297(TM1143). As it was observed that it is the charge of the methylation sites side chains, rather than their size or shape, that modulates kinase activity [29,30], the effect of methylation and demethylation was mimicked by modeling the side chains of residues corresponding to possible methylation sites with Qa beads (charged particle, hydrogen acceptor) in the demethylated system and with P1 beads (uncharged polar particle, low polarity) in the methylated system.

The obtained MD trajectories were analyzed as described in the Supporting Information S1 File.

## Supporting Information

S1 Fig The successive steps of the trimer-of-dimers model preparation. A: Initial model of the assembled dimer; B: The dimer in the simulation water box after 500 ns equilibration; C: Initial CG model of the trimer-of-dimers.
(TIF)

S2 Fig Relative longitudinal shift of the two helices in the inter-HAMP region during the equilibration simulation of the all-atom model of the *Np*HtrII dimer. (TIF)

S3 Fig Membrane curvature trajectories calculated for the methylated and the demethylated systems. (TIF)

S4 Fig Conformational effects of methylation/demethylation on the transmembrane region. Cytoplasmic view of a monomer of the *Np*SRII/*Np*HtrII complex (*left*). *Right top*: The shift of helix F at the cytoplasmic side with respect to the *Np*SRII protein (positive values correspond to an outward motion, blue arrow). *Right middle*: The average rotation angle of the TM2 helix with respect to the equilibrated methylated structure (positive values correspond to a clockwise rotation from the cytoplasmic side view, beige arrow). *Right bottom*: The average shift of the TM2 helix with respect to the TM1 helix of the transducer (positive values correspond to a shift towards the cytoplasm, green arrow).
(TIF)

S5 Fig Phase stutters in the HAMP domains. First (A) and second HAMP (B) domain of *Np*HtrII in comparison to the EcTsr HAMP domain. C: Location of the glycine-rich region in *Np*HtrII.
(TIF)

S6 Fig Inter-helical shifts in the tip region of *Np*HtrII. A: Inter-monomer shift averaged over the three dimers of the complex. B: Inter-helical shift between the two helices in the monomer averaged for the six monomers of the complex.
(TIF)

S7 Fig Inter-dimeric distances for the residues of the transducer. A: Inter-dimeric distances were calculated as an average over the three dimers for the methylated (black) and demethylated (red) states. The distance is measured between the center of mass (COM) of two related residues in one dimer and the COM of the six respective residues in the trimer-of-dimers (see B). The domains of the complex are depicted in colored bars; m.s and A/W indicate methylation sites and binding sites for CheA/CheW, respectively. Selected distance trajectories averaged over the regions of the transducer indicated by labeled rectangular boxes in A are depicted in figures C to G.
(TIF)

S8 Fig Length of the trimer of dimers in the methylated and the demethylated states. The length was measured between the center of mass of residues 23–83 and that if residues 352–372 of the transducer.
(TIF)

S9 Fig Relative shift of the two helices in the inter-HAMP region. The relative longitudinal shift is plotted for the three dimers (broken, dotted, and continuous lines) of the trimer-of-dimers for two independent CG MD simulations for the methylated (blue) and the demethylated (red) system.
(TIF)

S10 Fig Relative shift of the two helices in the inter-HAMP region during the demethylated-to-methylated transition. The relative longitudinal shift is plotted for three dimers (broken, dotted, and continuous lines) of the trimer-of-dimers. The depicted trajectory corresponds to the 1 μs-long simulation starting from the equilibrated demethylated system, in which the methylation state was swapped to the fully methylated one.
(TIFF)

S11 Fig Coiled-coil packing transitions in the methylation site region of the *Np*HtrII transducer dimer. Side-chain crick angles (as defined in [87]) are provided for the amphipathic sequence 2 of the second HAMP domain and for the N-terminal helix of the cytoplasmic domain. The angles were calculated from the equilibrium all-atom MD trajectory of the dimer and averaged over two monomers of the dimer. Values of 0 degrees correspond to complementary “x-da” packing, whereas deviating values (20–30 degrees) correspond to canonical “da” (knobs-into-holes) packing. The gray line depicts the behavior of ΔRMSF as calculated from the CG MD trajectories of the methylated and the demethylated trimer according to the data given in Fig 6. Zero crossings of ΔRMSF (red dotted lines) coincide with the change in packing mode.
(TIF)

S12 Fig RMSD plots calculated over the equilibration trajectory of the all-atom model. RMSD values for the transmembrane part (A), for the first HAMP domain (B), for the inter-HAMP region (C), for the second HAMP domain (D), and for the cytoplasmic domain (E) of the *Np*SRII/*Np*HtrII dimer.
(TIFF)

S13 Fig Two alternative oligomeric conformations for the *Np*SRII/*Np*HtrII trimer-of-dimers. A: Two equilibrated CG conformations of the trimer of dimers, the “triangle”-like conformation and the “ring”-like conformation based on results of docking shown in B and homology modeling of the inter receptor contacts according to the bacteriorhodopsin trimer. B: All atom representation of the “triangle”-like and “ring”-like conformations according to first- and the second-ranked predictions by the M-ZDOCK server [73,74]. C: RMSD between the two models shown in A. The RMSD was calculated for the transducer only (all six chains are averaged). For the transmembrane part of the complex RMSD values are obviously very large, however for the cytoplasmic domain the RMSD is generally less than 10 Å indicating close similarity between the two models in this key signal transduction region.
(TIF)

S14 Fig Time evolution of the conformation in the transmembrane region of the *Np*SRII/*Np*HtrII complex during the CG MD simulation of the demethylated-to-methylated transition. A: The shift of helix F at the cytoplasmic side with respect to the *Np*SRII protein. B: The rotation angle of helix TM2 with respect to the equilibrated methylated structure. C: The shift of helix TM2 with respect to helix TM1 of the transducer. D: Inter-dimeric distances for the adaptation region of the transducer.
(TIFF)

S15 Fig RMSD plot for one of the *Np*SRII receptors in the CG model of the methylated trimer. The trajectory shows the data from the constrained relaxation CG MD (2 μs) and the following unconstrained CG MD (6 μs).
(TIF)

S16 Fig Projections of the equilibrium trajectories onto the first three PCA eigenvectors for the methylated and the demethylated systems. The cosine content is given in the figure.
(TIF)

S1 Table Summary of the quality of the homology models used to build the all-atom structure of the dimer of *Np*HtrII. (PDF)

S2 Table Sequence alignment. The two regions of the *Ec*Tsr cytoplasmic domain shown contain the experimentally found methylation sites (red) [53] and are related to the corresponding regions of *Np*HtrII and *TM*1143, the latter was used as a structural template for homology model of the cytoplasmic domain of *Np*HtrII. Experimentally found methylation regions in *TM*1143 are highlighted (red) with the underlined residue indicating methylation at Gln-to-Glu substitution in mutant receptors [86]. Putative methylation sites used in the present study are colored in blue. At the bottom of the Table the consensus sequence for the methyltransferase CheR reported for enteric bacteria is shown [88].
(PDF)

S1 File Analysis of the MD trajectories. (PDF)
