# Supplementary figures and images for: Signaling and Adaptation Modulate the Dynamics of the Photosensoric Complex of Natronomonas pharaonis
Source: PLoS Comput Biol. 2015 Oct 23;11(10):e1004561. doi: 10.1371/journal.pcbi.1004561 (PMC4651059; doi:10.1371/journal.pcbi.1004561)

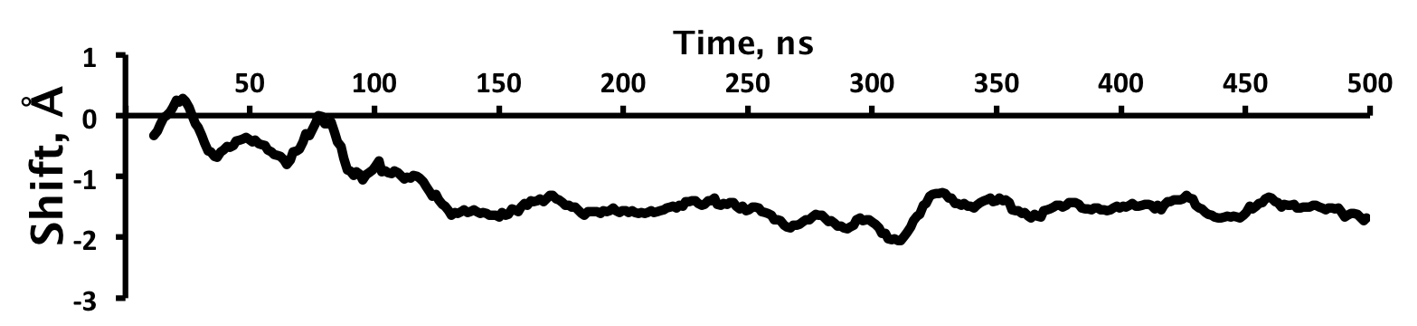

Supplement: S2 Fig — (TIF) [file pcbi.1004561.s002.tif]

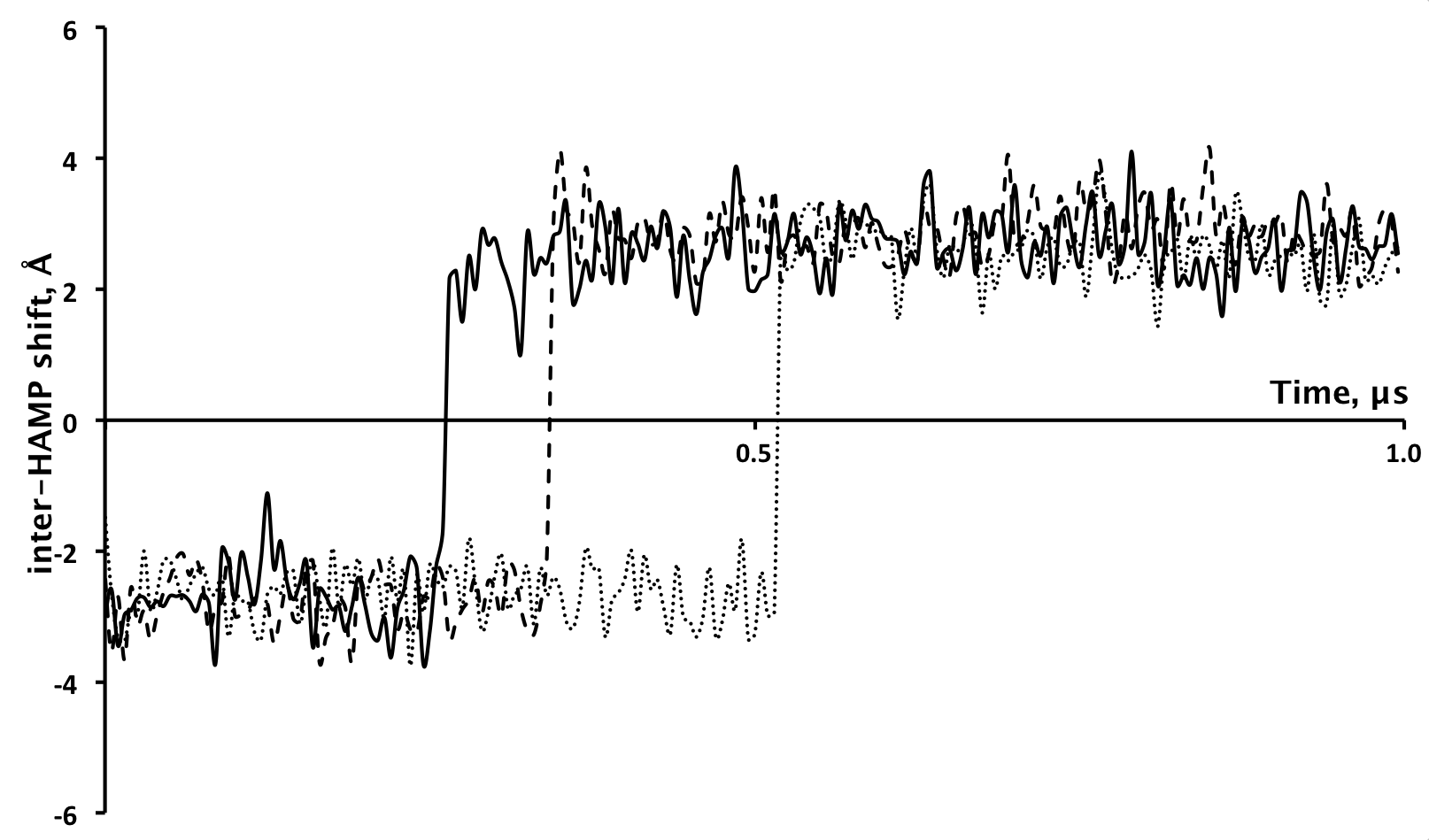

Supplement: S10 Fig — The relative longitudinal shift is plotted for three dimers (broken, dotted, and continuous lines) of the trimer-of-dimers. The depicted trajectory corresponds to the 1 μs-long simulation starting from the equilibrated demethylated system, in which the methylation state was swapped to the fully methylated one. (TIFF) [file pcbi.1004561.s010.tiff]

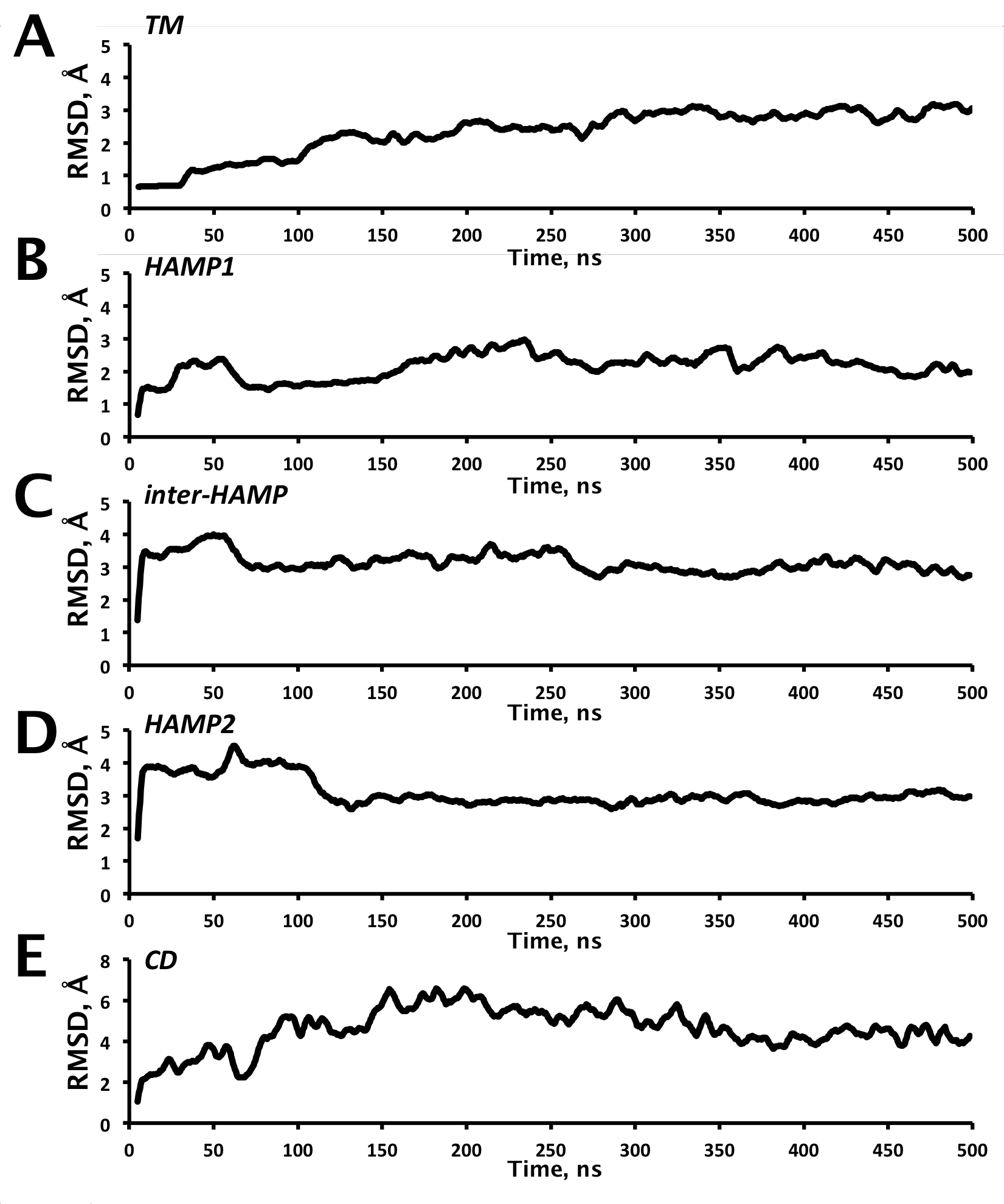

Supplement: S12 Fig — RMSD values for the transmembrane part (A), for the first HAMP domain (B), for the inter-HAMP region (C), for the second HAMP domain (D), and for the cytoplasmic domain (E) of the NpSRII/NpHtrII dimer. (TIFF) [file pcbi.1004561.s012.tiff]

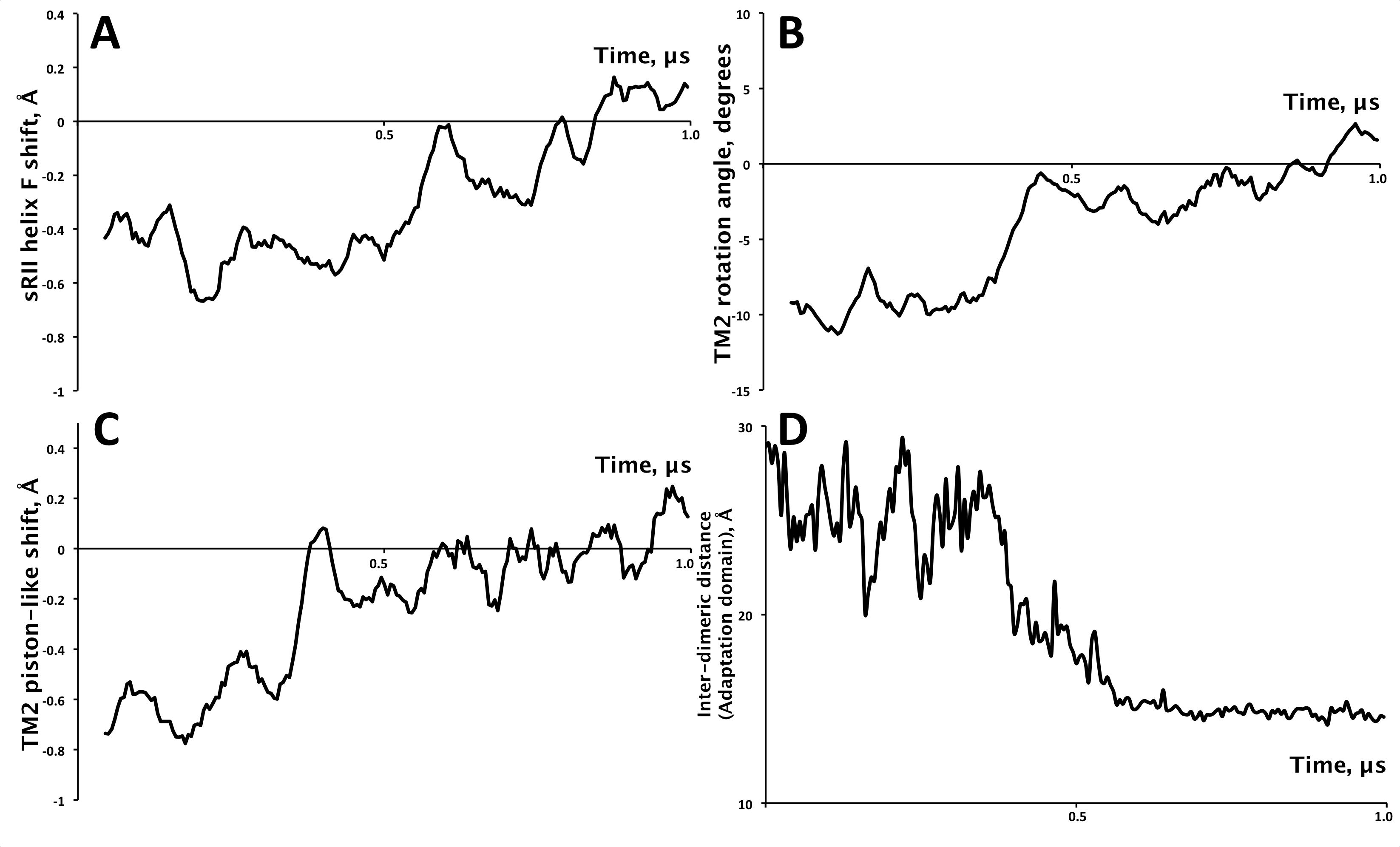

Supplement: S14 Fig — A: The shift of helix F at the cytoplasmic side with respect to the NpSRII protein. B: The rotation angle of helix TM2 with respect to the equilibrated methylated structure. C: The shift of helix TM2 with respect to helix TM1 of the transducer. D: Inter-dimeric distances for the adaptation region of the transducer. (TIFF) [file pcbi.1004561.s014.tiff]

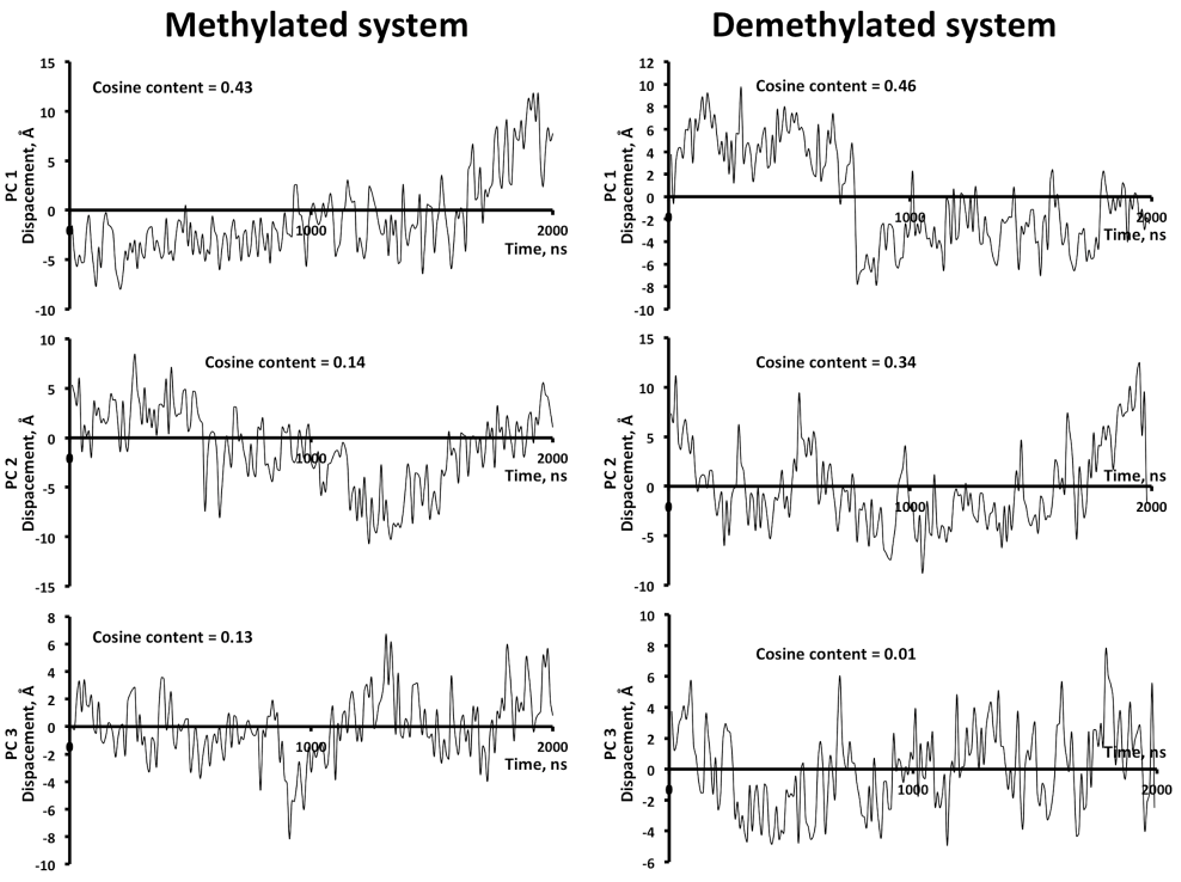

Supplement: S16 Fig — The cosine content is given in the figure. (TIF) [file pcbi.1004561.s016.tif]
