# Supplementary material for: Signaling and Adaptation Modulate the Dynamics of the Photosensoric Complex of Natronomonas pharaonis
Source: PLoS Comput Biol. 2015 Oct 23;11(10):e1004561. doi: 10.1371/journal.pcbi.1004561 (PMC4651059; doi:10.1371/journal.pcbi.1004561)
Supplement: S1 Table — (PDF) [file pcbi.1004561.s017.pdf]

S1 Table. Summary of the quality of the homology models used to build the all-atom structure of the dimer of *Np*HtrII.

Model	Template	Sequence identity in %	Sequence similarity in %	Template DOPE score	Model DOPE score
Cytoplasmic domain	2CH7	24.7	64.7	-69482	-58921
HAMP1	2ASW	25.9	75.9	-11712	-3817
HAMP2	2ASW	7.3	58.2	-11712	-3737
