# Supplementary material for: Signaling and Adaptation Modulate the Dynamics of the Photosensoric Complex of Natronomonas pharaonis
Source: PLoS Comput Biol. 2015 Oct 23;11(10):e1004561. doi: 10.1371/journal.pcbi.1004561 (PMC4651059; doi:10.1371/journal.pcbi.1004561)
Supplement: S2 Table — The two regions of the EcTsr cytoplasmic domain shown contain the experimentally found methylation sites (red) [53] and are related to the corresponding regions of NpHtrII and TM1143, the latter was used as a structural template for homology model of the cytoplasmic domain of NpHtrII. Experimentally found methylation regions in TM1143 are highlighted (red) with the underlined residue indicating methylation at Gln-to-Glu substitution in mutant receptors [86]. Putative methylation sites used in the present study are colored in blue. At the bottom of the Table the consensus sequence for the methyltransferase CheR reported for enteric bacteria is shown [88]. (PDF) [file pcbi.1004561.s018.pdf]

```
htr2    :  258 VQQIALEMDDVSATTEEVAAS 278
tsr     :  296 ---------EQQAASLEETAAS 308
tm1143  :  273 VEETTAGSEEEISSATKNIADS 293

htr2    :  464 MAATSEQTASDAET 477
tsr     :  480 MDRVTQQNAALVEE 493
tm1143  :  479 NAKNAEEITNSVKE 492

Consensus: (A/S)XXEEX(A/T/S)A(A/T/S)
```
