# Supplementary material for: Signaling and Adaptation Modulate the Dynamics of the Photosensoric Complex of Natronomonas pharaonis
Source: PLoS Comput Biol. 2015 Oct 23;11(10):e1004561. doi: 10.1371/journal.pcbi.1004561 (PMC4651059; doi:10.1371/journal.pcbi.1004561)
Supplement: S1 File — (PDF) [file pcbi.1004561.s019.pdf]

Analysis of trajectories

The analysis of the trajectories was conducted in VMD [1] using its TCL scripting interface and with Python scripts using the ProDy package [2].

The length of the complexes was defined as a sum of the partial lengths (from the center of mass (COM) of the transmembrane part to the COM of the second HAMP domain and from the COM of the second HAMP domain to the COM of the tip region) due to a possible impairing influence of the bending of the protein complex during the simulation both in the methylated and the demethylated state. However, this bending motion did not affect other observables because of their local nature and relatively high period of this bending (μs time scale).

All of the measures stemming from the individual dimers were averaged over the three dimers in the trimer. Analogously, the measures of the monomers were averaged over all six monomers in the trimer. Prior to the RMSF calculations, the protein complexes were first aligned by the rigid transmembrane region comprising SRII and the transmembrane region of HtrII (res. 23 to 82).

The membrane curvature was calculated by dividing the membrane into three equal parts and the COMs of the phosphate groups of the lipids in each part were calculated. The inverse radius of the sphere drawn through these three points was considered as a curvature measure of the membrane in the selected direction. In order to obtain the total curvature, the values along the X- and Y-axis were averaged.

To verify that the observed dynamical changes are essentially of random nature and to thus justify the use of RMSF as a valid measure for the dynamics, we performed a principal component analysis [3] and calculated the cosine content for projections of the equilibrium trajectories onto the first three PCA eigenvectors [4, 5]. Despite the long simulation time, we found similar harmonic content in the first components, the cosine content being 0.43 and 0.46 for the methylated and the demethylated systems, respectively (see S16 Fig.). These results indicate the significant role of “random diffusion” degrees of freedom in the dynamics of the studied systems as well as the lack of large conformational rearrangements.

Reference List

1. Humphrey, W., Dalke, A., & Schulten, K. VMD: Visual molecular dynamics. *Journal of Molecular Graphics* **14**, 33-38 (1996).
2. Bakan, A., Meireles, L.M., & Bahar, I. ProDy: Protein Dynamics Inferred from Theory and Experiments. *Comput. Appl. Biosci.* **27**, 1575-1577 (2011).
3. Hayward, S. & Go, N. Collective Variable Description of Native Protein Dynamics. *Annu. Rev. Phys. Chem.* **46**, 223-250 (1995).
4. Hess, B. Similarities between principal components of protein dynamics and random diffusion. *Phys. Rev. E* **62**, 8438-8448 (2000).
5. Hess, B. Convergence of sampling in protein simulations. *Phys. Rev. E* **65**, 031910 (2002).
